# Electrolyte-Regulated Ion Accommodation and Microstructural Stability in WO_3_ Electrochromic Thin Films

**DOI:** 10.3390/mi17091033

**Published:** 2026-08-29

**Authors:** Xuefeng Chu, Siming Qiao, Wenhao Ma, Kunjie Lin, Faxin Peng, Xinyuan Zhang, Jie Wu, Haiyang Zhao, Longyu Guo, Huan Wang, Sa Lv, Xiaotian Yang

**Affiliations:** 1 Key Laboratory of Architectural Cold Climate Energy Management, Ministry of Education, Jilin Jianzhu University, Changchun 130118, China; 13153807860@163.com (S.Q.); 19963936313@163.com (W.M.); linkunjie1@163.com (K.L.); 18574368242@163.com (F.P.); 17550296224@163.com (X.Z.); wanghuan@jlju.edu.cn (H.W.); lvsa@jlju.edu.cn (S.L.); 2 School of Electrical and Computer Science, Jilin Jianzhu University, Changchun 130118, China; 3 Department of Chemistry, Jilin Normal University, Siping 136000, China; jiewu@jlnu.edu.cn (J.W.); hanyxt@163.com (X.Y.); 4 Electron Microscopy Center, Jilin University, Changchun 130012, China; guolongyu@jlu.edu.cn

**Keywords:** WO_3_ thin films, electrochromism, ion accommodation, electrolyte regulation, electronic structure evolution, microstructural stability, ion insertion/extraction

## Abstract

Electrochromic tungsten oxide (WO_3_) films suffer from gradual performance degradation during repeated ion insertion/extraction processes, while the relationship between electrolyte-dependent ion accommodation and structural stability remains insufficiently understood. Herein, magnetron-sputtered WO_3_ thin films were systematically investigated in H_2_SO_4_, LiClO_4_, ZnSO_4_, and Al_2_(SO_4_)_3_ electrolytes to clarify the coupling relationship among ion accommodation, electronic structure evolution, microstructural retention, and electrochromic durability. Combined electrochemical measurements with UV–visible spectroscopy, atomic force microscopy (AFM), scanning electron microscopy (SEM), X-ray photoelectron spectroscopy (XPS), and reflected electron energy loss spectroscopy (REELS) reveal that electrolyte chemistry regulates the balance between electrochemical activation and structural tolerance. Highly mobile H^+^ and strongly interacting Al^3+^ ions promote rapid ion transport and enhanced W^6+^ reduction but may induce excessive structural perturbation, defect accumulation, and accelerated degradation during cycling. Zn^2+^ exhibits intermediate behavior, whereas Li^+^ enables balanced ion accommodation through reversible W^6+^/W^5+^ conversion while preserving the WO_3_ framework. Consequently, the LiClO_4_ electrolyte achieves superior electrochromic performance with an optical modulation of 80.97% and improved cycling stability. These results demonstrate that durable electrochromic behavior is governed not by maximizing ion transport or reduction degree, but by achieving a reversible ion accommodation regime compatible with the structural tolerance of the host framework. This work provides a microstructure-oriented strategy for designing stable WO_3_-based electrochromic devices through electrolyte regulation.

## 1. Introduction

Electrochromic (EC) technologies have attracted increasing attention for applications in smart windows, adaptive optical devices, low-energy displays, and thermal management systems owing to their capability of reversibly regulating optical transmission under external electrical stimuli [1,2,3,4]. With the transition of electrochromic devices from laboratory demonstrations toward practical applications, achieving long-term operational stability has become as important as obtaining large optical modulation and fast switching kinetics [5,6,7,8]. However, the durability of many electrochromic systems remains limited by irreversible structural evolution during repeated ion insertion and extraction processes [9,10,11]. Therefore, understanding how electrochemical reactions induce microstructural changes and eventually determine device degradation has become a critical scientific issue for the development of reliable electrochromic materials [12,13]. Tungsten trioxide (WO_3_) is one of the most extensively investigated inorganic electrochromic materials because of its large optical modulation, excellent chemical stability, and compatibility with scalable deposition techniques [13,14,15,16]. It should be noted that WO_3_ electrochromic films can exist in either crystalline or amorphous states depending on the preparation conditions. Compared with crystalline WO_3_, amorphous WO_3_ possesses a disordered open framework with abundant structural flexibility, which facilitates reversible ion accommodation during electrochemical cycling. However, the absence of long-range structural ordering also makes amorphous WO_3_ more susceptible to local structural rearrangement, defect accumulation, and stress-induced degradation under repeated ion insertion/extraction processes. Therefore, understanding ion accommodation and microstructural stability in amorphous WO_3_ frameworks is critical for developing durable electrochromic devices [17]. The electrochromic behavior of WO_3_ originates from coupled ion-electron insertion processes, in which guest ions enter the WO_3_ framework accompanied by electron injection and the partial reduction of tungsten species from W^6+^ to W^5+^ [15,16,17,18]. Although ion insertion activates the optical switching process, repeated insertion/extraction inevitably induces local structural rearrangement, lattice strain accumulation, and defect evolution [10,16]. Consequently, the same process that enables electrochromism may also become the origin of irreversible microstructural degradation. This fundamental trade-off raises an important question: how can ion insertion efficiently activate electrochromic switching while preserving the structural integrity of the WO_3_ framework?

Electrolyte engineering provides an effective approach to regulate ion transport and interfacial reactions in electrochromic systems [18]. Different electrolyte ions exhibit distinct characteristics, including ionic radius, charge density, diffusion kinetics, and ion–framework interaction strength, which can significantly influence ion accommodation behavior within WO_3_ [17,18,19]. Proton-based electrolytes generally provide rapid switching kinetics because of the high mobility of H^+^ [20], while lithium-based systems are often associated with improved cycling stability due to moderate ion insertion and relatively weak lattice perturbation [21]. Recently, multivalent ions such as Zn^2+^ and Al^3+^ have received increasing attention because their higher charge density enables efficient charge compensation and strong electrochemical activity. However, excessive interaction between multivalent ions and oxide frameworks may generate pronounced lattice distortion, interfacial stress, and irreversible structural reconstruction [17,18,19,20,21,22]. Proton-based electrolytes generally provide rapid switching due to high H^+^ mobility, whereas lithium-based electrolytes often exhibit improved cycling stability owing to moderate ion insertion and reduced structural perturbation. Multivalent ions such as Zn^2+^ and Al^3+^ provide enhanced charge compensation but may introduce stronger ion–framework interactions and structural instability. However, current studies mainly focus on electrolyte optimization based on optical performance and switching kinetics, while the relationship among ion characteristics, electronic-state evolution [23], and microstructural degradation remains insufficiently understood [24,25].

In this work, we introduce the concept of balanced ion accommodation to describe the coupling relationship among ion transport capability, electronic modulation, and structural tolerance of the WO_3_ framework [26]. Unlike conventional electrolyte screening strategies that mainly aim to maximize electrochemical activity, this concept emphasizes achieving a reversible ion accommodation regime that enables optical activation while maintaining structural stability. Four representative electrolytes (H_2_SO_4_, LiClO_4_, ZnSO_4_, and Al_2_(SO_4_)_3_) were systematically investigated using electrochemical measurements combined with UV–visible spectroscopy, AFM, SEM, XPS, and REELS analyses. The results reveal that highly mobile H^+^ and strongly interacting Al^3+^ ions accelerate electrochemical activation but promote structural degradation, whereas Li^+^ provides a more favorable balance between reversible ion accommodation and framework preservation. This work provides a microstructure-oriented strategy for understanding electrolyte-dependent ion insertion behavior and designing durable WO_3_-based electrochromic systems.

## 2. Experimental Section

### 2.1. Preparation of WO_3_ Thin Films

Commercial indium tin oxide (ITO) conductive glass substrates (Nuozhuo Technology Co., Ltd., Hangzhou, China) were used as transparent conductive substrates for WO_3_ thin-film deposition. The ITO substrates possessed dimensions of 20 mm × 10 mm × 1.1 mm, a sheet resistance of 7–10 Ω sq^−1^, an ITO thickness of 185 ± 50 nm, and a surface roughness of approximately 3 nm.

Prior to deposition, a conductive copper foil was attached to the edge region of the ITO substrate to ensure reliable electrical contact during subsequent electrochemical measurements. WO_3_ thin films were deposited by reactive magnetron sputtering using a tungsten oxide target. The deposition process was performed under an Ar_2_/O_2_ atmosphere with a gas flow ratio of 18:2, a working pressure of 1.4 Pa, a sputtering power of 100 W, and a deposition time of 15 min. The target-to-substrate distance was maintained at 12 cm. The thickness was maintained comparable among all samples because identical deposition parameters were used.

Based on our previous structural analysis under identical sputtering conditions, these WO_3_ films are considered amorphous [20] and were subsequently employed as model electrochromic structures to investigate electrolyte-dependent ion accommodation behavior and its influence on microstructural evolution during electrochemical cycling.

### 2.2. Selection and Preparation of Electrolytes

To clarify the relationship between ion characteristics and microstructural evolution of WO_3_ films, four representative electrolyte systems with different ion valence states and transport behaviors were selected, including H_2_SO_4_, LiClO_4_, ZnSO_4_, and Al_2_(SO_4_)_3_.

Among these electrolytes, H_2_SO_4_ provides highly mobile proton insertion, LiClO_4_ introduces monovalent Li^+^ ions with moderate interaction strength, whereas Zn^2+^ and Al^3+^ represent multivalent ions with stronger electrostatic interactions with the WO_3_ framework. The effective ionic radii of the inserted species (H^+^, Li^+^, Zn^2+^, and Al^3+^) are approximately 0.53, 0.76, 0.74, and 0.54 Å, respectively. These differences in ionic size, together with their valence states and charge densities, determine the ion–framework interaction strength and consequently influence ion accommodation behavior and structural stability during cycling [27].

The LiClO_4_ electrolyte was a commercial 1.0 mol L^−1^ LiClO_4_/propylene carbonate (PC) solution purchased from Kelude New Energy Technology Co., Ltd., Dongguan, China. The H_2_SO_4_, ZnSO_4_, and Al_2_(SO_4_)_3_ electrolytes were prepared by dissolving analytical-grade reagents in ultrapure water with a concentration of 1.0 mol L^−1^. Sulfuric acid was obtained from Jingji Scientific Reagent, while zinc sulfate and aluminum sulfate were supplied by Sinopharm Chemical Reagent Co., Ltd., Shanghai, China.

All electrolytes were prepared under identical concentration conditions to minimize concentration-dependent effects and allow direct comparison of ion-induced structural evolution in WO_3_ films [28].

### 2.3. Electrochemical Characterization and Ion Intercalation Analysis

Electrochemical measurements were conducted using a CHI760E electrochemical workstation (Shanghai Chenhua Instrument Co., Ltd., Shanghai, China) with a conventional three-electrode configuration. The WO_3_ film deposited on ITO glass served as the working electrode, a platinum sheet was used as the counter electrode, and Ag/AgCl was employed as the reference electrode [29].

Cyclic voltammetry (CV) measurements were performed to analyze the ion insertion/extraction behavior and electrochemical reversibility of WO_3_ films in different electrolyte environments. The scan rates ranged from 0.01 to 0.10 V s^−1^ within a potential window of −1.0 to 1.0 V. The relationship between peak current density and the square root of the scan rate was analyzed to evaluate the apparent ion transport behavior.

Chronoamperometric (CA) measurements were carried out to investigate the evolution of electrochemical reversibility during repeated coloration/bleaching processes. A square-wave voltage of ±1.0 V was applied with a duration of 20 s for each coloration and bleaching step.

The electrochemical measurements were combined with morphological and surface chemical analyses to establish the correlation between ion transport behavior, microstructural evolution, and electrochromic stability [30].

### 2.4. Structural and Chemical Characterization

The surface morphology and cross-sectional microstructure of the WO_3_ thin films were characterized using a field-emission scanning electron microscope (SEM, JSM-7900F, JEOL Ltd., Tokyo, Japan). The surface morphology and roughness were further investigated using an atomic force microscope (AFM, MFP-3D Infinity, Oxford Instruments, Oxford, UK). The chemical composition, elemental valence states, chemical bonding configurations, and reflected electron energy loss spectra (REELS) were characterized using an X-ray photoelectron spectrometer (XPS, ESCALAB 250 Xi, Thermo Fisher Scientific, Waltham, MA, USA).

## 3. Results

### 3.1. Electrolyte-Dependent Microstructural Evolution of WO_3_ Films

**Figure 1:**
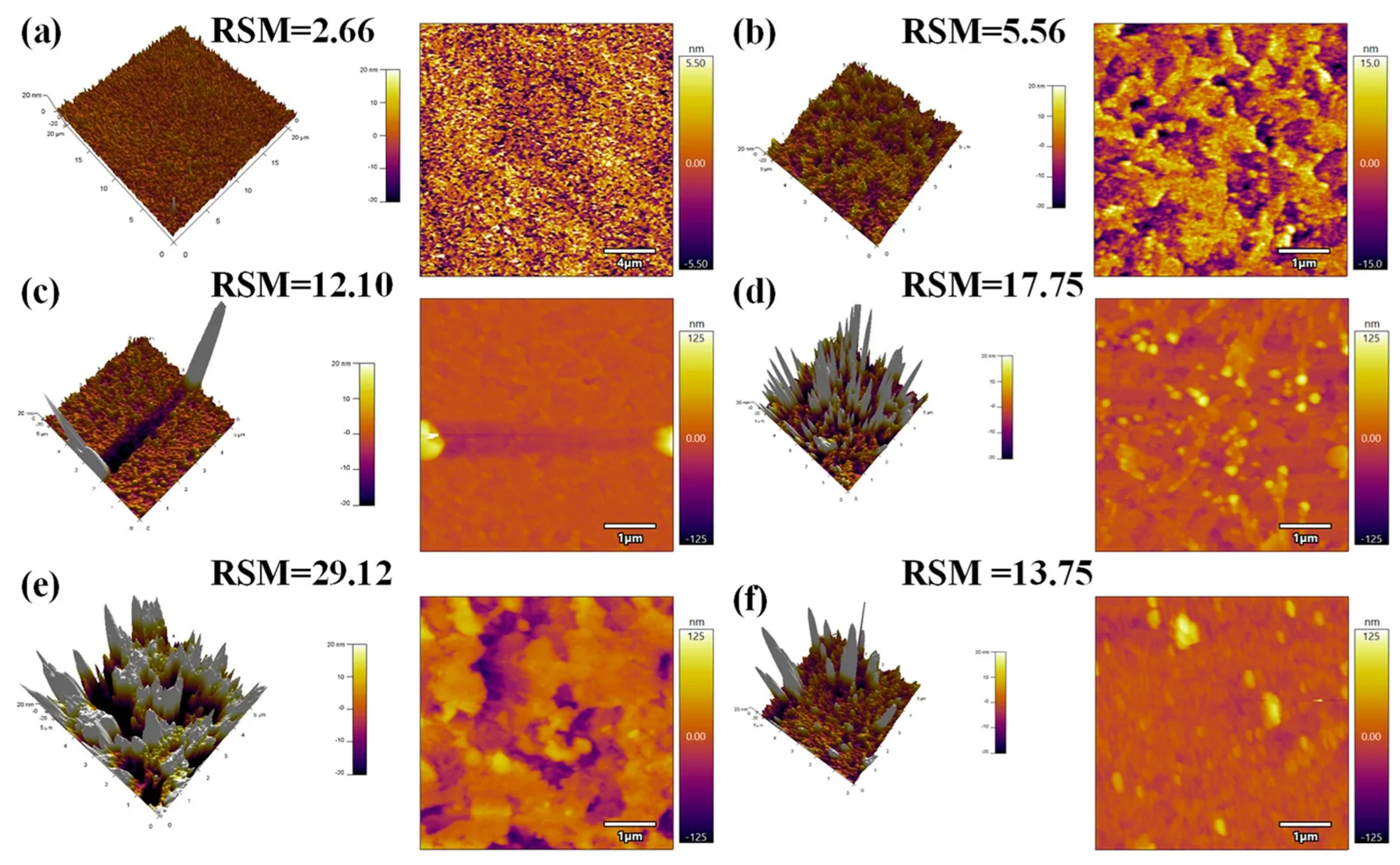
AFM characterization of the surface morphology evolution of WO_3_ thin films in different electrolytes during electrochemical cycling. (**a**) Bare ITO substrate. (**b**) Pristine WO_3_ thin film before electrochemical cycling. (**c**) WO_3_ thin film after 20 coloration/bleaching cycles in the H_2_SO_4_ electrolyte. (**d**) WO_3_ thin film after 20 coloration/bleaching cycles in LiClO_4_ electrolyte. (**e**) WO_3_ thin film after 20 coloration/bleaching cycles in the ZnSO_4_ electrolyte. (**f**) WO_3_ thin film after 20 coloration/bleaching cycles in the Al_2_(SO_4_)_3_ electrolyte.

**Figure 2:**
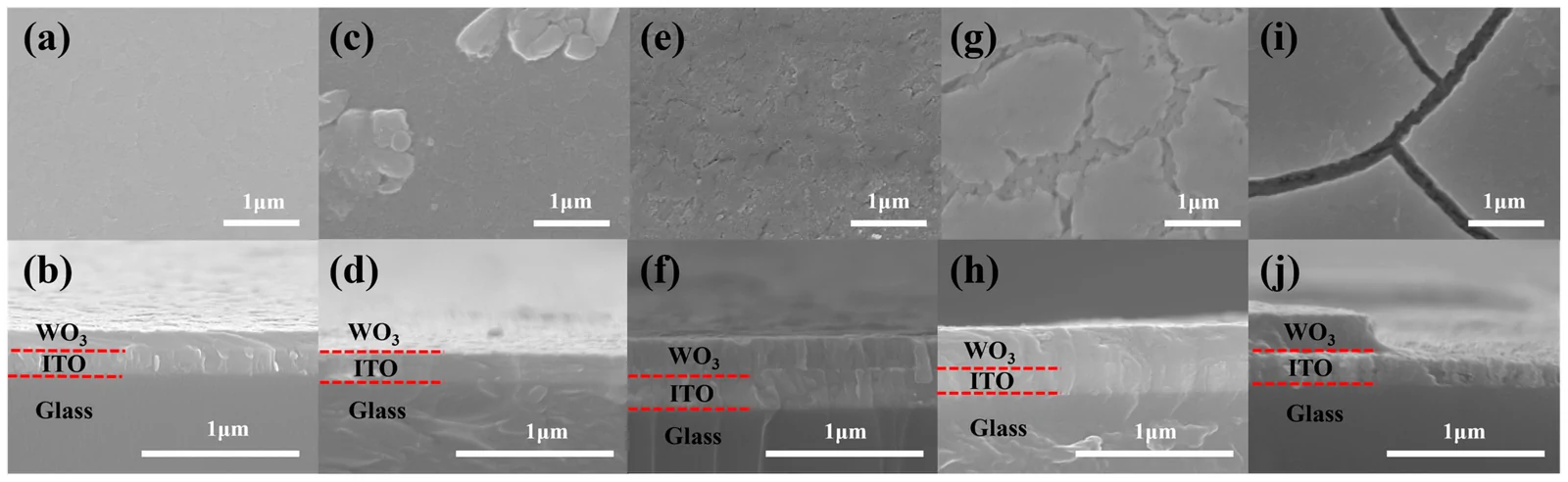
Electrolyte-dependent evolution of the surface morphology and cross-sectional microstructure of WO_3_ thin films after 20 electrochemical coloration/bleaching cycles. (**a**) Surface morphology of the pristine WO_3_ thin film. (**b**) Cross-sectional morphology of the pristine WO_3_ thin film. (**c**) Surface morphology after cycling in the H_2_SO_4_ electrolyte. (**d**) Cross-sectional morphology after cycling in the H_2_SO_4_ electrolyte. (**e**) Surface morphology after cycling in the LiClO_4_ electrolyte. (**f**) Cross-sectional morphology after cycling in the LiClO_4_ electrolyte. (**g**) Surface morphology after cycling in the ZnSO_4_ electrolyte. (**h**) Cross-sectional morphology after cycling in the ZnSO_4_ electrolyte. (**i**) Surface morphology after cycling in the Al_2_(SO_4_)_3_ electrolyte. (**j**) Cross-sectional morphology after cycling in the Al_2_(SO_4_)_3_ electrolyte.

The microstructural stability of WO_3_ films during repeated ion insertion/extraction processes was first investigated by AFM and SEM characterization. As shown in Figure 1 and Figure 2, the pristine WO_3_ film exhibits a compact and continuous surface morphology with uniform coverage on the ITO substrate, indicating the successful formation of a stable electrochromic layer by magnetron sputtering.

After 20 coloration/bleaching cycles in different electrolyte environments, pronounced differences in microstructural evolution are observed. The WO_3_ films cycled in H_2_SO_4_ and Al_2_(SO_4_)_3_ electrolytes exhibit significant surface roughening, crack formation, and partial film detachment [31]. These phenomena indicate that rapid ion insertion or strong ion–framework interaction can induce irreversible structural reconstruction during repeated electrochemical cycling [32].

In contrast, the LiClO_4_-treated WO_3_ film maintains a relatively continuous surface morphology with limited structural deterioration, while the ZnSO_4_ system shows moderate surface degradation. These results demonstrate that electrolyte-dependent structural evolution is not simply determined by ion insertion capability but is closely associated with the ability of the WO_3_ framework to accommodate inserted ions. Therefore, maintaining a suitable balance between ion insertion and structural tolerance is essential for preserving the integrity of WO_3_ electrochromic films [33].

### 3.2. Ion Accommodation Behavior and Transport–Structure Correlation

To understand the relationship between ion transport behavior and microstructural stability, cyclic voltammetry measurements were performed at different scan rates. All WO_3_ films exhibit reversible redox responses corresponding to ion insertion/extraction coupled with W^6+^/W^5+^ conversion.

**Figure 3:**
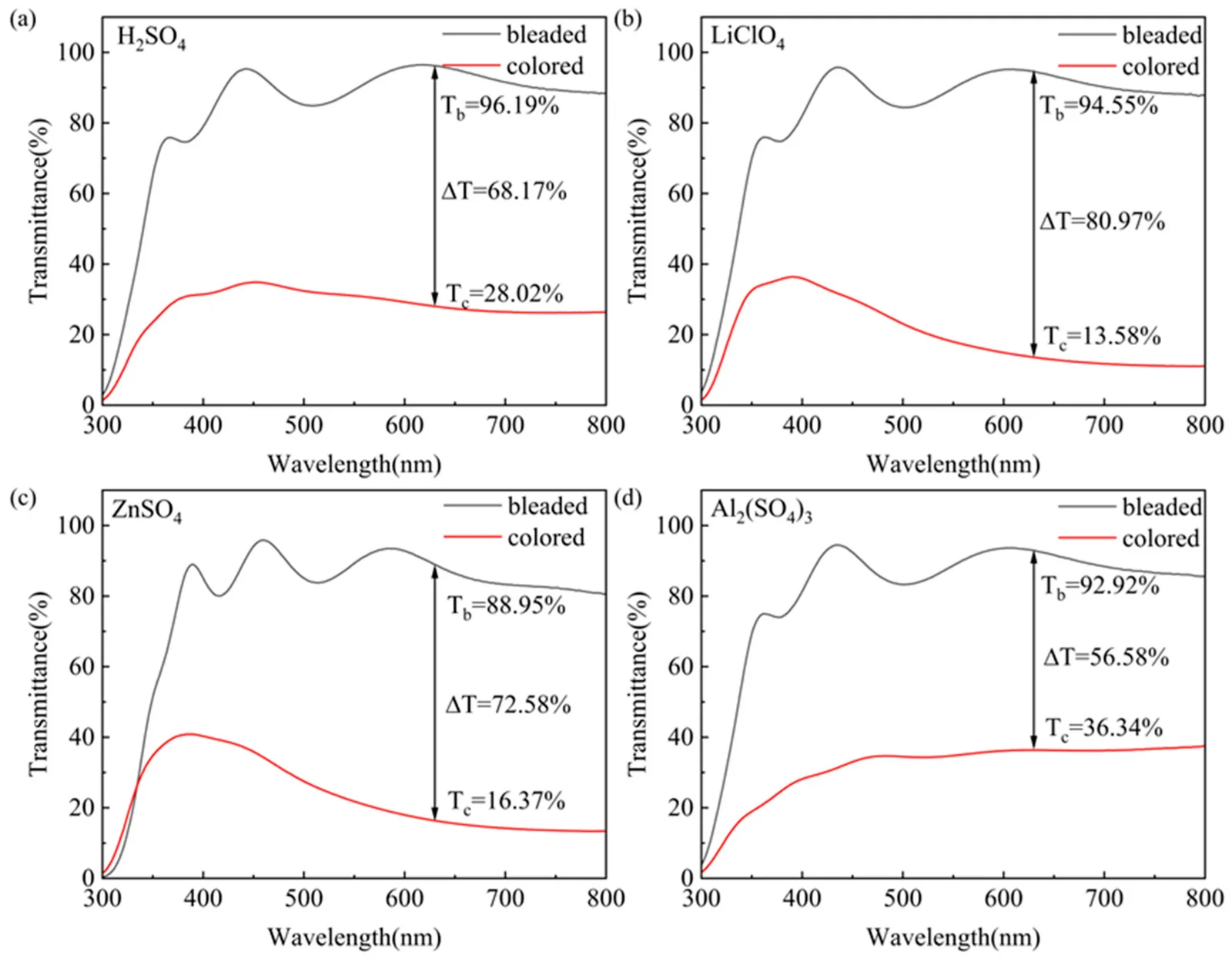
Optical modulation behavior of WO_3_ films regulated by electrolyte-dependent structural evolution. Transmittance spectra of WO_3_ films during coloration and bleaching processes in (**a**) H_2_SO_4_, (**b**) LiClO_4_, (**c**) ZnSO_4_, and (**d**) Al_2_(SO_4_)_3_.

**Figure 4:**
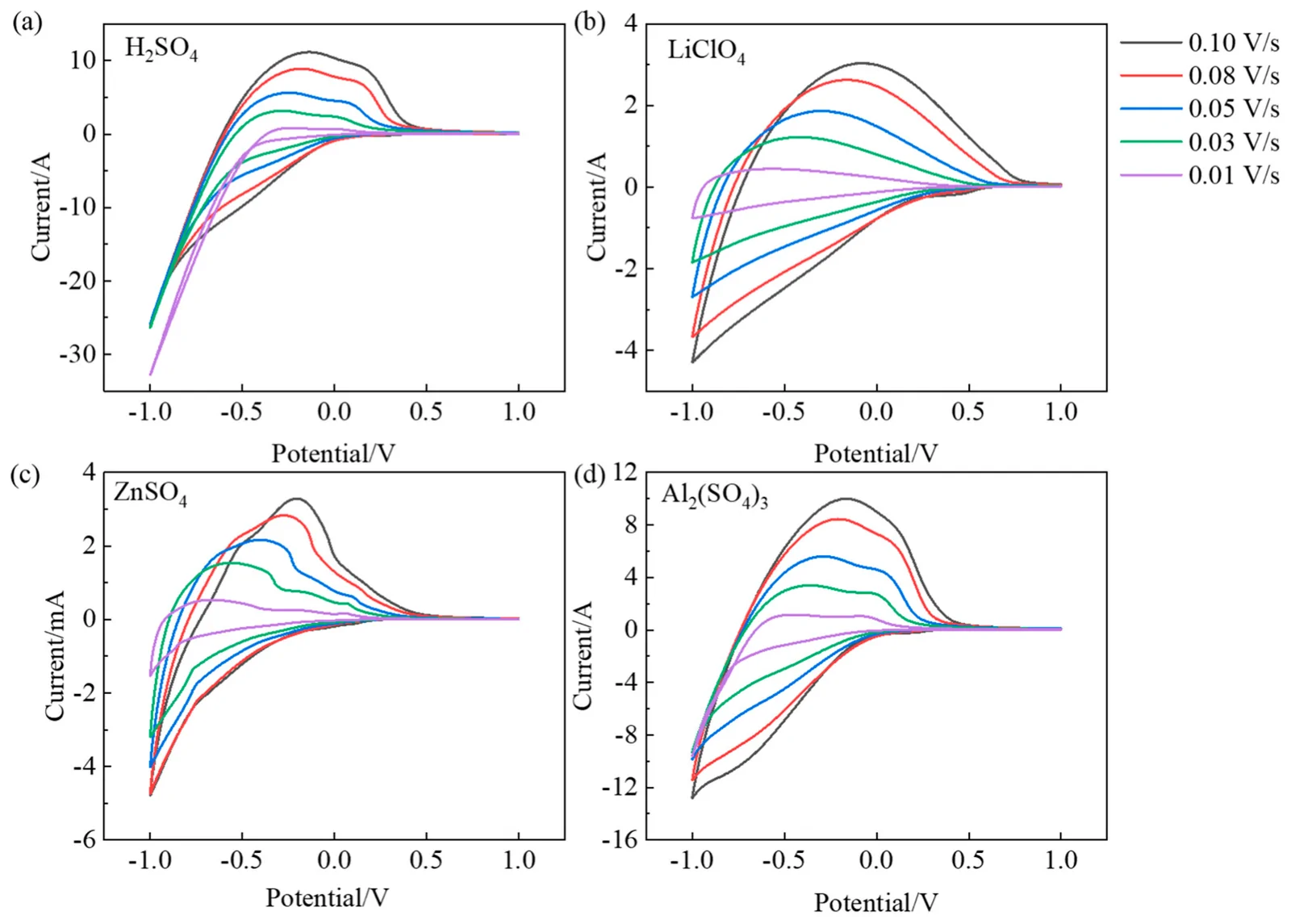
Ion insertion/extraction kinetics of WO_3_ films under different electrolyte environments. Cyclic voltammograms of WO_3_ films recorded at different scan rates in (**a**) H_2_SO_4_, (**b**) LiClO_4_, (**c**) ZnSO_4_, and (**d**) Al_2_(SO_4_)_3_.

**Figure 5:**
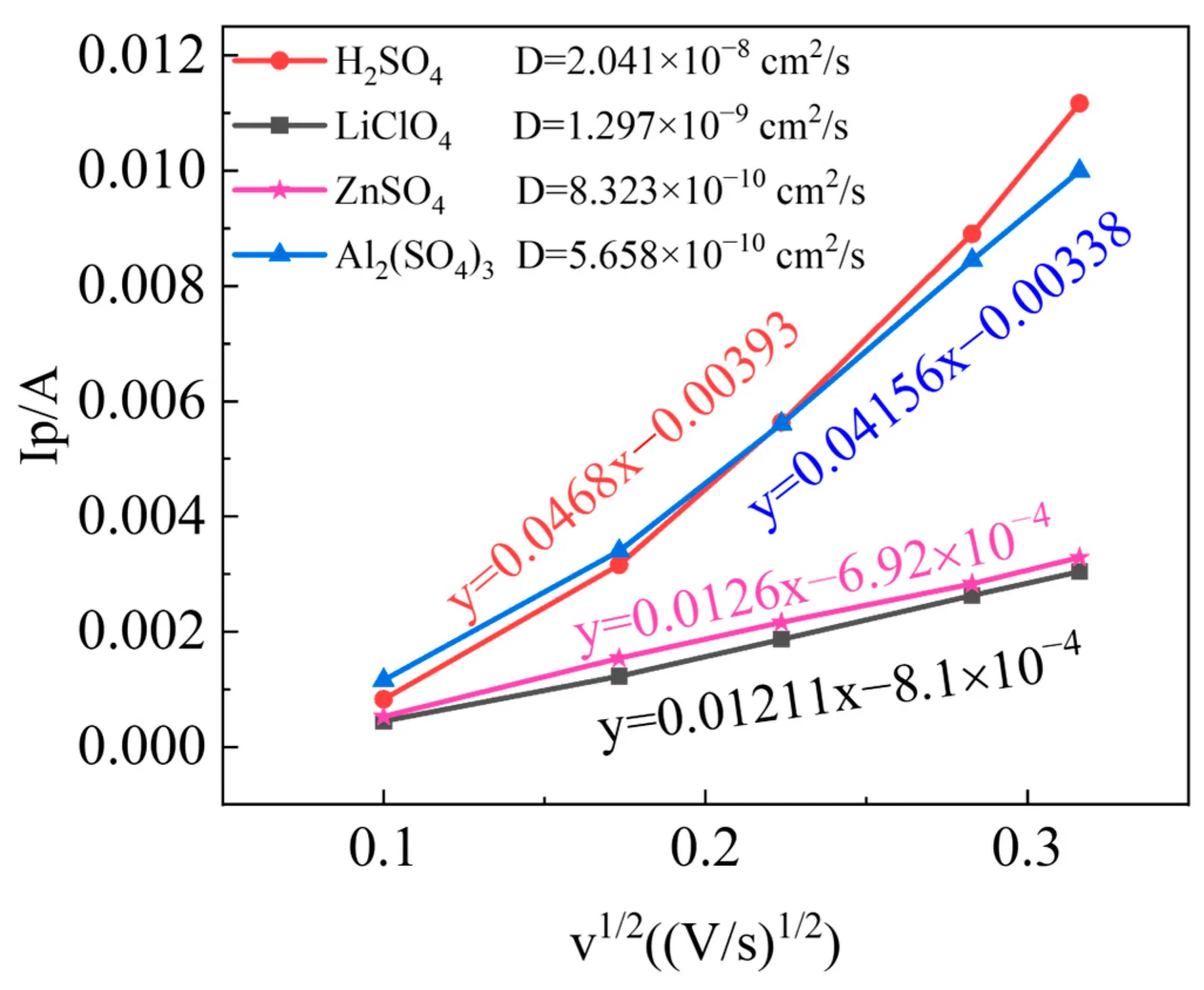
Correlation between ion transport kinetics and electrochemical response of WO_3_ films. Linear relationship between peak current density and square root of scan rate for WO_3_ films operated in different electrolyte systems.

The optical modulation behaviors of WO3 films in different electrolytes are first compared in Figure 3. Although LiClO4 exhibits the highest optical modulation, the underlying ion transport behavior requires further analysis. Therefore, cyclic voltammetry and diffusion analyses were performed, as shown in Figure 4 and Figure 5.

However, the ion transport capability does not directly correlate with structural stability. Although H^+^ and Al^3+^ provide faster electrochemical responses, the corresponding WO_3_ films suffer from pronounced morphological degradation after cycling. Conversely, Li^+^ exhibits moderate transport behavior but maintains superior structural integrity.

This apparent contradiction indicates that rapid ion transport alone is insufficient to achieve durable electrochromic performance. Instead, the interaction between inserted ions and the host WO_3_ framework determines whether ion insertion proceeds through reversible structural accommodation or irreversible reconstruction [34].

### 3.3. Microstructural Retention Governs Electrochemical Cycling Stability and Electrochromic Durability

The relationship between ion accommodation behavior, microstructural evolution, and electrochromic durability was further evaluated through optical modulation and electrochemical cycling measurements. As shown in Figure 5, all WO_3_ films exhibit reversible coloration and bleaching responses under different electrolyte environments, confirming effective ion insertion/extraction processes. However, significant differences in optical modulation are observed among the electrolytes. The LiClO_4_ electrolyte achieves the highest optical modulation of 80.97%, followed by ZnSO_4_ (72.58%), whereas H_2_SO_4_ and Al_2_(SO_4_)_3_ show relatively lower modulation despite their strong electrochemical activity. This discrepancy indicates that enhanced ion insertion capability does not necessarily translate into improved electrochromic performance.

To further clarify this phenomenon, cyclic voltammetry measurements were conducted to evaluate the ion insertion/extraction kinetics. As shown in Figure 4, all electrolyte systems display reversible redox characteristics associated with the coupled insertion of guest ions and reduction of W^6+^ to W^5+^.

The apparent ion transport kinetics was further analyzed by correlating peak current density with the square root of scan rate (Figure 5). The calculated diffusion-related slopes reveal that different electrolyte systems possess distinct ion transport capabilities. Notably, the order of ion transport kinetics does not coincide with the electrochromic stability trend. Although H^+^ and Al^3+^ provide faster ion transport, the corresponding WO_3_ films do not maintain superior electrochromic performance during repeated operation.

**Figure 6:**
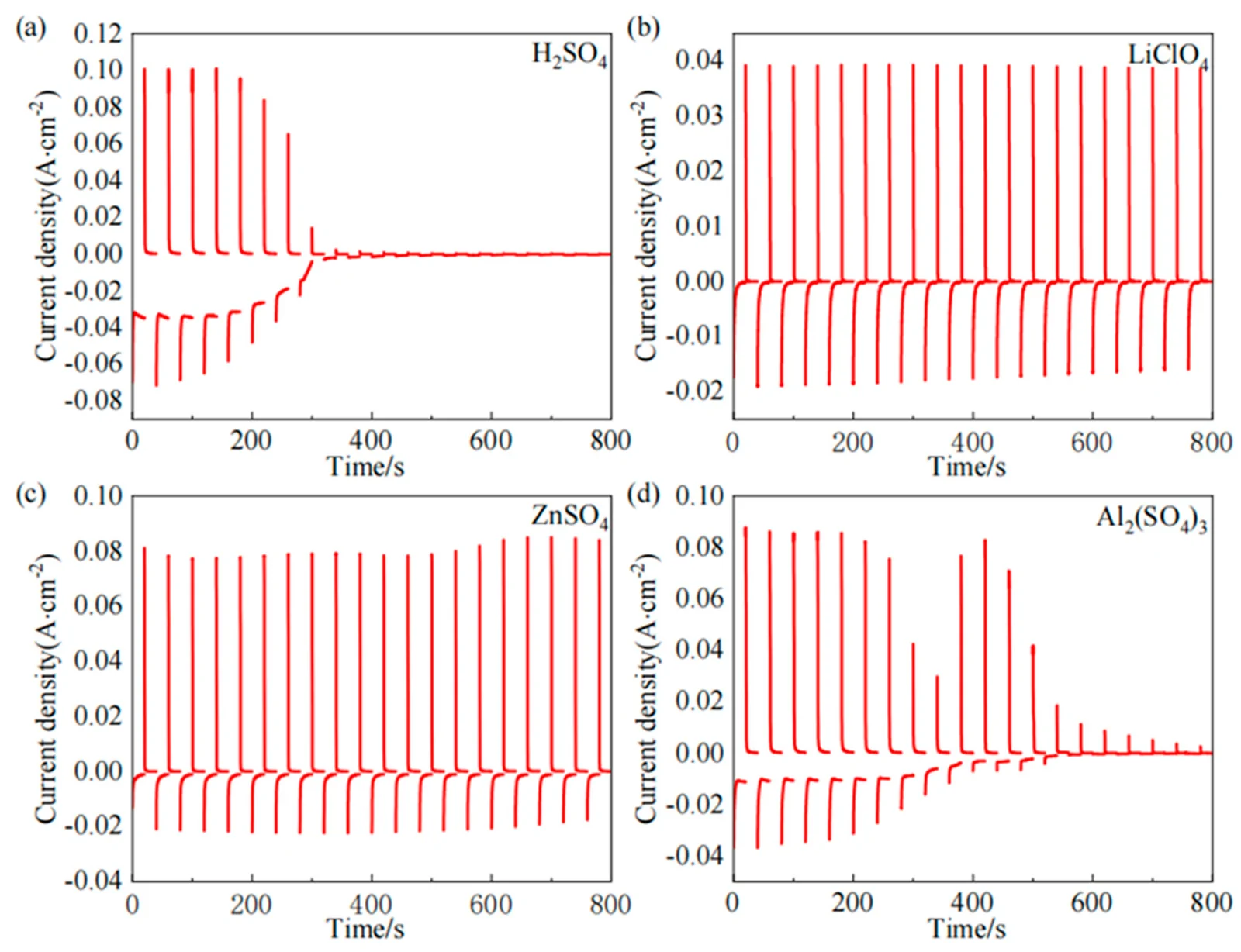
Electrochemical cycling stability associated with microstructural retention of WO_3_ films. Chronoamperometric responses of WO_3_ films during repeated coloration/bleaching cycles in different electrolytes: (**a**) H_2_SO_4_, (**b**) LiClO_4_, (**c**) ZnSO_4_, and (**d**) Al_2_(SO_4_)_3_.

To further evaluate the influence of electrolyte-dependent structural evolution on cycling stability, chronoamperometric measurements were performed during repeated coloration/bleaching processes (Figure 6). All samples exhibit periodic anodic and cathodic current responses, confirming reversible ion insertion/extraction behavior. However, significant differences in current retention are observed among different electrolyte systems. The H_2_SO_4_ electrolyte exhibits the highest initial current response due to rapid proton insertion; nevertheless, the current density decreases continuously during cycling, indicating progressive degradation of electrochemically active regions. Similarly, the Al_2_(SO_4_)_3_ electrolyte shows pronounced current attenuation, which can be attributed to the strong interaction between multivalent Al^3+^ ions and the WO_3_ lattice, resulting in irreversible structural reconstruction and reduced reversibility [35].

In contrast, the LiClO_4_ electrolyte maintains highly stable anodic and cathodic current responses throughout repeated cycling, demonstrating excellent reversibility of Li^+^ insertion/extraction processes. The ZnSO_4_ electrolyte exhibits intermediate behavior, with moderate current retention and structural stability. These cycling characteristics are consistent with the morphological observations obtained from AFM and SEM analyses (Figure 1 and Figure 2), where LiClO_4_-treated WO_3_ films preserve a relatively intact surface morphology, whereas H_2_SO_4_- and Al_2_(SO_4_)_3_-treated films experience severe roughening and crack formation.

Collectively, the results from Figure 3, Figure 4, Figure 5 and Figure 6 demonstrate that electrochromic durability is not governed by maximum ion transport rate or initial electrochemical activity, but rather by the ability of the WO_3_ framework to accommodate repeated ion insertion within a reversible structural regime. Therefore, maintaining a balance between ion mobility and structural tolerance is essential for achieving stable and durable WO_3_-based electrochromic systems.

### 3.4. Electronic Structure Evolution Associated with Electrolyte-Dependent Ion Accommodation

**Figure 7:**
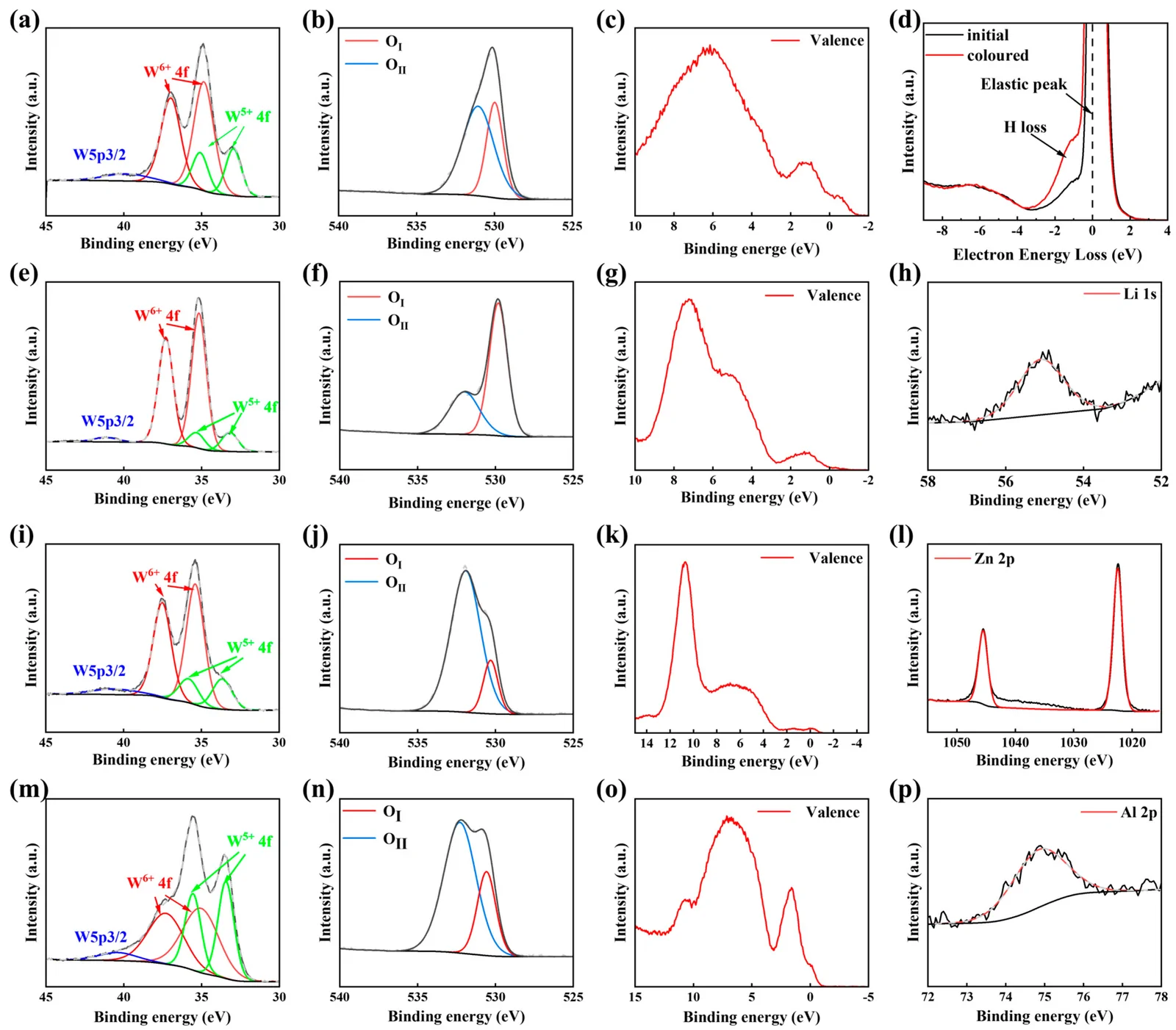
Electrolyte-dependent electronic structure evolution of WO_3_ films during ion insertion. (**a**–**d**) W 4f, O 1s, valence band, and REELS spectra of the WO_3_ film colored in H_2_SO_4_ electrolyte; (**e**–**h**) W 4f, O 1s, valence band, and Li 1s spectra of the WO_3_ film colored in LiClO_4_ electrolyte; (**i**–**l**) W 4f, O 1s, valence band, and Zn 2p spectra of the WO_3_ film colored in ZnSO_4_ electrolyte; (**m**–**p**) W 4f, O 1s, valence band, and Al 2p spectra of the WO_3_ film colored in the Al_2_(SO_4_)_3_ electrolyte.

To further elucidate the electronic origin underlying the electrolyte-dependent electrochromic behavior, X-ray photoelectron spectroscopy (XPS) and reflected electron energy loss spectroscopy (REELS) measurements were performed to investigate the electronic structure evolution of WO_3_ films after ion insertion. As shown in Figure 7, all electrolyte systems exhibit characteristic changes associated with the electrochromic coloration process, confirming that ion insertion is coupled with electron injection and partial reduction of tungsten species from W^6+^ to W^5+^ [36].

The W 4f spectra (Figure 7a,e,i,m) reveal clear variations in the relative contribution of W^5+^ and W^6+^ components among different electrolyte environments. The increased W^5+^ fraction after coloration confirms that inserted ions provide charge compensation through coupled electron transfer, which is responsible for the formation of colored tungsten oxide states. However, the extent of tungsten reduction differs significantly depending on the inserted ion species. The H_2_SO_4_ and Al_2_(SO_4_)_3_ electrolytes induce stronger W^5+^ generation, indicating enhanced electron compensation and more intensive electrochemical activation. Nevertheless, excessive tungsten reduction may introduce a higher concentration of reduced states and defect-related electronic structures, which agrees with the severe morphological degradation and rapid electrochemical decay observed in Figure 1, Figure 2 and Figure 6.

In contrast, the LiClO_4_ electrolyte produces a moderate increase in W^5+^ species while maintaining a relatively balanced W^6+^/W^5+^ ratio. This controlled electronic-state modulation suggests that Li^+^ insertion provides sufficient electron compensation to activate optical switching without inducing excessive structural perturbation. Such a balanced reduction process contributes to the superior optical modulation and cycling stability observed in the LiClO_4_ system.

The O 1s spectra (Figure 7b,f,j,n) further provide information regarding the evolution of oxygen-related chemical environments during ion insertion. The variation in lattice oxygen and defect-related oxygen components indicates that different electrolyte systems induce distinct degrees of oxygen framework perturbation. Electrolytes producing stronger ion–framework interactions, particularly H_2_SO_4_ and Al_2_(SO_4_)_3_, lead to more pronounced oxygen-related structural changes, suggesting increased defect formation during repeated ion accommodation. Conversely, Li^+^ insertion causes relatively limited modification of the oxygen coordination environment, indicating improved preservation of the WO_3_ framework.

The valence band spectra (Figure 7c,g,k,o) provide additional insight into the relationship between electronic structure and electrochromic performance. After coloration, all samples exhibit enhanced electronic states near the valence band edge, corresponding to the generation of reduced tungsten species and increased electronic states associated with W^5+^ formation. The H_2_SO_4_ and Al_2_(SO_4_)_3_ systems show stronger electronic-state modification, consistent with their higher reduction degree and stronger initial electrochemical responses. However, excessive electronic-state evolution is accompanied by irreversible structural changes, resulting in reduced cycling stability. In comparison, LiClO_4_ induces moderate valence-band modification, indicating sufficient electronic activation while avoiding excessive defect-state generation. This balanced electronic modulation provides an explanation for the enhanced optical modulation and durability of Li^+^-based electrochromic systems.

Furthermore, REELS spectra (Figure 7d) provide additional evidence regarding hydrogen-related ion accommodation. Since hydrogen has a low atomic mass and cannot be reliably detected by conventional XPS analysis, REELS was employed to qualitatively identify hydrogen-related electronic loss features associated with H^+^ insertion/extraction. The observed hydrogen-related energy-loss signals confirm the participation of proton insertion in the H_2_SO_4_ electrolyte system, providing direct spectroscopic evidence for proton-mediated coloration. The rapid appearance and disappearance of these features during electrochemical operation further support the high mobility of H^+^ ions, which explains the fast electrochromic response but also the enhanced structural stress accumulation during repeated cycling [37].

Overall, Figure 7 demonstrates that electrolyte-dependent electrochromic behavior originates from the coupled regulation of ionic accommodation and electronic structure evolution. This behavior is associated with their high charge density and strong interaction with the WO_3_ framework. In contrast, Li^+^ with a moderate effective ionic radius and lower charge density enables reversible ion accommodation with reduced lattice perturbation. In contrast, Li^+^ enables moderate electronic modulation through reversible W^6+^/W^5+^ conversion while maintaining the structural integrity of the WO_3_ framework. These findings further confirm that durable electrochromic performance is achieved not by maximizing ion-induced electronic activation, but by balancing electronic modulation with structural tolerance during repeated ion insertion/extraction processes.

### 3.5. Mechanistic Understanding of Electrolyte-Regulated Ion Accommodation and Microstructural Stability

Based on the combined results of electrochemical measurements, microstructural characterization, and electronic structure analysis, an electrolyte-regulated ion accommodation mechanism is proposed to elucidate the relationship among ion transport behavior, structural evolution, and electrochromic durability of WO_3_ films.

The electrochromic process of WO_3_ involves coupled ion insertion and electron compensation, where inserted ions enter the oxide framework accompanied by the reduction of W^6+^ to W^5+^. However, the accommodation behavior of different ions within the WO_3_ lattice determines whether this process proceeds through reversible structural adaptation or irreversible reconstruction. Therefore, the electrochromic stability is not solely determined by ion mobility or reduction capability, but by the balance between electrochemical activation and structural tolerance.

For the H_2_SO_4_ electrolyte system, highly mobile H^+^ ions promote rapid ion transport and strong electrochemical responses, as demonstrated by the CV and chronoamperometric results. The enhanced proton insertion induces pronounced W^5+^ formation and strong electronic modulation observed from XPS and valence-band spectra. However, the excessive proton accommodation generates accumulated structural stress and defect evolution, leading to surface roughening, crack formation, and accelerated electrochemical decay during cycling. Therefore, although H^+^ provides fast switching kinetics, the limited structural tolerance of the WO_3_ framework restricts long-term durability.

A similar but more pronounced structural limitation is observed in the Al_2_(SO_4_)_3_ electrolyte. The high charge density of Al^3+^ ions enables strong electrostatic interaction with the WO_3_ framework, resulting in intensive electron compensation, enhanced W^5+^ generation, and significant modification of electronic states. However, the strong ion–lattice interaction is likely associated with stronger local structural perturbation and irreversible reconstruction tendency, which accounts for the rapid deterioration of electrochemical reversibility and reduced electrochromic stability.

The ZnSO_4_ electrolyte exhibits an intermediate ion accommodation behavior. Zn^2+^ provides moderate electrochemical activity and structural perturbation, resulting in better cycling retention compared with H^+^ and Al^3+^ systems, but the ion–framework interaction is still insufficiently optimized to achieve the highest electrochromic durability.

In contrast, LiClO_4_ provides an optimized ion accommodation environment. The moderate interaction strength and suitable transport behavior of Li^+^ enable reversible W^6+^/W^5+^ conversion while minimizing excessive lattice distortion and defect accumulation. This balanced electronic modulation is reflected by moderate W^5+^ generation, controlled valence-band evolution, the preserved oxygen coordination environment, and REELS characteristics. Consequently, the Li^+^-based WO_3_ film maintains an intact microstructure, ion insertion/extraction pathways, and superior optical modulation and cycling durability.

Overall, the results demonstrate that durable electrochromic performance originates from a balanced ion accommodation process rather than maximizing ion insertion kinetics or electronic reduction degree. Excessive ion accommodation accelerates defect formation and structural degradation, whereas insufficient electrochemical activation limits optical response. Achieving an appropriate balance between ion mobility, electronic modulation, and structural tolerance enables reversible electrochemical transformation and long-term stability of WO_3_ electrochromic films. This ion accommodation-regulated microstructural evolution mechanism provides a general strategy for designing durable electrochromic materials through electrolyte engineering.

## 4. Conclusions

In this work, the relationship between electrolyte-dependent ion accommodation, microstructural evolution, electronic structure regulation, and electrochromic durability of magnetron-sputtered WO_3_ thin films was systematically investigated using four representative electrolyte systems (H_2_SO_4_, LiClO_4_, ZnSO_4_, and Al_2_(SO_4_)_3_). By combining electrochemical characterization with morphological and spectroscopic analyses, the mechanism governing the activity–stability trade-off during ion insertion/extraction was elucidated.

The results demonstrate that electrolyte chemistry strongly determines the accommodation behavior of inserted ions within the WO_3_ framework. Although H^+^ and Al^3+^ induce faster ion transport, stronger tungsten reduction, and more pronounced electronic-state modulation, excessive ion accommodation generates structural stress, defect accumulation, and irreversible microstructural degradation. These effects lead to accelerated electrochemical decay and reduced long-term electrochromic stability. Zn^2+^ exhibits moderate interaction with the WO_3_ amorphous network and achieves an intermediate balance between electrochemical activity and structural preservation.

In contrast, Li^+^ insertion provides an optimized accommodation environment, enabling reversible W^6+^/W^5+^ conversion while maintaining the structural integrity and ion insertion pathways of the WO_3_ framework. The superior optical modulation (80.97%) and cycling stability achieved in the LiClO_4_ electrolyte originate from the balanced interaction among ion transport capability, electronic modulation, and structural tolerance rather than from the highest ion mobility or reduction degree.

Based on these findings, an electrolyte-regulated ion accommodation mechanism is proposed, demonstrating that durable electrochromic performance requires reversible structural adaptation during repeated ion insertion/extraction processes. This study provides a microstructure-oriented strategy for understanding and designing retained WO_3_-based electrochromic systems through controlled ion–framework interactions. Beyond electrochromic applications, the regulation of ion accommodation and framework tolerance may also provide useful insights into tungsten oxide-based electrochemical energy-storage systems involving reversible ion insertion and structural evolution [38,39]. More broadly, the relationships among defect regulation, electronic structure, and structural stability are also relevant to other WO_3_-based technologies, including photocatalysis [40,41,42] and solar-energy applications [43].

## Data Availability

The data supporting the findings of this study are available from the corresponding author upon reasonable request.
